# Incorporation of functional elements enhances the antitumor capacity of CAR T cells

**DOI:** 10.1186/s40164-017-0088-z

**Published:** 2017-10-11

**Authors:** Le Qin, Ruocong Zhao, Peng Li

**Affiliations:** 1 Key Laboratory of Regenerative Biology, South China Institute for Stem Cell Biology and Regenerative Medicine, Guangzhou Institutes of Biomedicine and Health, Chinese Academy of Sciences, Guangzhou, 510530 China; 2 Guangdong Provincial Key Laboratory of Stem Cell and Regenerative Medicine, South China Institute for Stem Cell Biology and Regenerative Medicine, Guangzhou Institutes of Biomedicine and Health, Chinese Academy of Sciences, Guangzhou, 510530 China; 3 State Key Laboratory of Respiratory Disease, Guangzhou Institutes of Biomedicine and Health, Chinese Academy of Sciences, Guangzhou, 510530 China

**Keywords:** CAR T cells, Tumor microenvironment, Cytokines, Immune checkpoint molecules, Hypoxia, Chemotherapeutic drugs, Kinase inhibitors

## Abstract

As chimeric antigen receptor (CAR) T cells have displayed an unprecedented efficacy in the treatment of CD19-positive malignances, it is believed that this cell therapy will be a milestone in the history of mankind’s conquering of cancer. However, there are some issues that restrict CAR T cells from reaching their optimal anti-tumor capacity, especially in the treatment of solid tumors. Inhibitory cytokines, immune checkpoint molecules, hypoxia and other adverse factors have been reported to be involved in this process. To obtain better efficacy in the treatment of leukemia and solid tumors, we need to continuously upgrade CAR T cell technology by incorporating novel functional elements into CAR T cells to overcome these restrictions. In this review, we summarize recent advances regarding this topic.

## Background

**Fig. 1:**
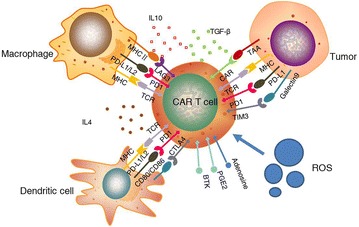
Adverse factors including inhibitory cytokines, immune checkpoint molecules and hypoxia restrict CAR T cells from reaching an optimal anti-tumor capacity in the tumor microenvironment

Benefiting from the remarkable therapeutic outcome of chimeric antigen receptor (CAR) T cell treatment of refractory/relapsed B cell malignancy, CAR T cells are transitioning from the lab to the cancer ward. Several CAR-T products are in the advanced stage of clinical development [1–3]. CARs are recombinant receptors that generally consist of scFv, hinge, transmembrane domain, costimulatory molecule and CD3 ζ chain. Current research on CAR T cells mainly focuses on exploring new scFvs that are expressed at high levels on tumor cell surfaces rather than in healthy tissue and investigating an appropriate co-stimulatory intensity that enhances CAR T cell killing capacity and persistence [4–6]. However, there are some issues that restrict CAR T cells from reaching an optimal anti-tumor capacity, especially in the treatment of solid tumors [7]. Such as inhibitory cytokines, immune checkpoint molecules, hypoxia and other adverse factors hindering CAR T cell from efficiently expanding and killing tumor cells. Some researchers are attempting to combine some novel elements into CAR vectors to make CAR T cell slip the leash of aforementioned restrictions and thereby exhibiting optimal antitumor capacity (Fig. 1). In this review, we summarize the progresses of the incorporation of novel functional elements to improve CAR T cells, which have obtained prominent results.

## Cytokines

IL-12 is a heterodimeric inflammatory cytokine produced by activated antigen-presenting cells (APCs), neutrophils, and macrophages. Scientific research has shown that a hostile tumor microenvironment can be significantly regulated by IL-12 through multiple mechanisms, including reactivation of anergic tumor-infiltrated lymphocytes (TILs), inhibition of Treg-mediated suppression of effector T cells, recruitment of NK cells to the tumor site, and inhibition of IL-10 and transforming growth factor-β (TGF-β) secretion by tumor-associated macrophages (TAM). In a novel syngeneic tumor model, Pegram et al. [8] demonstrated that tumor elimination requires both CD4+ and CD8+ T cell subsets, autocrine IL-12 stimulation, and subsequent IFN-γ secretion by CAR T cells. Therefore, they modified CD19-targeted CAR T cells to constitutively secrete IL-12, and the results showed that this treatment was able to safely eradicate established disease in the absence of prior conditioning and to acquire intrinsic resistance to Treg-mediated inhibition. You et al. [9] modified CAR T cell targeting MUC1 antigen to co-express IL12 for the treatment of seminal vesicle cancer in a phase I clinical trial. Interestingly, they found that the co-expression of IL-12 may contribute to TIA-1 expression in tumors after MUC1 CAR T cell treatment. IL-4 is an immunosuppressive cytokine in the tumor microenvironment that can promote fibrogenesis, support tumor growth and protect malignant cells from immune destruction. To protect CAR-PSCA T cells from the inhibitory effects of IL4, Somala Mohammed transgenically expressed a custom inverted cytokine receptor (ICR) in which the IL-4 receptor ectodomain was fused to the IL-7 receptor endodomain, switching the inhibitory effects to promoting effects to ultimately result in potent and sustained anti-tumor effects [10]. To achieve the selective expansion of CAR T cells, Whilding et al. [11] co-expressed an IL-4-responsive fusion gene (4αβ), which fused the IL-4 receptor α ectodomain to the shared human IL-2/IL-15 receptor β transmembrane and endodomain regions. Binding of IL-4 led to the delivery of a potent and selective growth signal in 4αβ+ CAR T cells.

## Immune checkpoint molecules

As an evasion mechanism, many tumors are able to express various immune checkpoint molecules, resulting in the exhaustion of T cells that cannot prevent tumor progression. Emerging clinical data have highlighted the importance of the PD-L1/PD-1 immune inhibitory axis, and immune checkpoint blockers targeting both PD1 and PD-L1 have obtained great success in cancer therapy [12, 13]. Suarez et al. [14] developed a new combination immunotherapy that consists of human anti-carbonic anhydrase IX (CAIX)-targeted CAR T cells engineered to secrete human antibodies at the tumor site. Local anti-PD-L1 antibody delivery led to a fivefold reduction in tumor growth and a 50–80% reduction in tumor weight when compared with the anti-CAIX CAR T cells alone in a humanized mice model of clear cell renal cell carcinoma (ccRCC). What was more interesting was that because the isotype of the anti-PD-L1 antibody was IgG1, it had the potential to mediate ADCC and was able to recruit NK cells to the tumor site. Tanoue et al. [15] designed a new immunotherapy strategy for the treatment of prostate cancer; this strategy comprised an oncolytic adenovirus (Onc.Ad), a helper-dependent adenovirus (HDAd) that expressed a PD-L1 blocking mini-antibody, and HER2.CAR T cells. The results demonstrated that this combinatory therapy enhanced the anti-tumor effect compared with treatment with either HER2.CAR T cells alone or with HER2.CAR T cells plus Onc.Ad, and the benefits of locally produced PD-L1 mini-body could not be replaced by the infusion of anti-PD-L1 IgG. To overcome PD-L1 immunosuppressive effects on adoptively transferred T cells, Prosser et al. [16] converted PD-1 to a T cell costimulatory receptor by substituting its transmembrane and intracellular domains with the CD28 domain. Rather than becoming exhausted upon engagement of PDL1+ tumors, adoptively transferred T cells modified to express this PD1:CD28 chimera exhibited enhanced functional attributes. In addition, several research groups have tried to rescue CAR T from exhaustion through gene editing. For example, Ren et al. [17] used the one-shot CRISPR protocol to generate allogeneic universal T cells, simultaneously editing four gene loci, to decrease the expression of both PD1 and CTLA-4. Liu et al. [18] also constructed PD1 knockout universal CAR T cells.

## Hypoxia

Hypoxia is a hallmark of a hostile tumor microenvironment, and tumor cells have high levels of oxidative stress and reactive oxygen species (ROS) production that substantially impair the antitumor activity of adoptively transferred T cells. Ligtenberg et al. [19] presented a strategy to render antitumor T cells more resilient toward ROS by co-expressing catalase along with a tumor-specific CAR to increase their anti-oxidative capacity by metabolizing H_2_O_2_. CAR T cells engineered to co-express catalase (CAR-CAT) showed increased levels of intracellular catalase and sharp decreases in ROS accumulation while maintaining their antitumor activity despite high H_2_O_2_ levels. More importantly, CAR T cells co-expressing catalase substantially protected bystander effector cells from oxidative stress-mediated repression. The lung is an oxygen-rich environment that frequently permits colonization by metastatic tumor cells. The prolyl hydroxylase domain (PHD) functions as an intracellular sensor of oxygen. Clever et al. [20] found that T cell intrinsic expression of PHD proteins maintained local tolerance against innocuous antigens in the lung. Meanwhile, PHD proteins also restrained the responses of the pulmonary-type helper Th-1 and CD8+ T cells, promoted Treg cell induction, and finally intensively enabled the colonization of circulating tumor cells. Inhibition of PHD proteins by dimethyloxalylglycine (DMOG) increased Th1 differentiation and IFN-γ production compared with vehicle-treated cultures. DMOG-treated TRP-1 CD4+ T cells mediated superior clearance of established subcutaneous tumors and lung metastases of melanoma. Based on the hypoxic tumor microenvironment, Juillerat et al. [21] developed an oxygen-sensitive CAR by introducing an oxygen-sensitive subdomain of HIF1α to a CAR scaffold. CAR begins to be expressed only when CAR T cells infiltrate a low oxygen environment, and expression is rapidly switched down once the cells get away from the hypoxic environment.

## Chemotherapeutic drugs and kinase inhibitors

Chemotherapeutic drugs and kinase inhibitors play important roles in clinical cancer treatment. In order to elicit stronger antitumor activity, some researchers are attempting to combine CAR T cell therapy with these drugs. Fraietta et al. [22] found that T cells from chronic lymphocytic leukemia (CLL) patients usually exhibit deficiencies in proliferation, and this proliferative defect is completely reversed after long-term ibrutinib therapy. Ibrutinib is a first-in-class irreversible inhibitor of Bruton tyrosine kinase (BTK), which can irreversibly inhibit the IL-2 inducible T cell kinase (ITK). Ibrutinib inhibits Th2-polarized CD4 T cells, thus skewing T cells toward a Th1 anti-tumor immune response. Therefore, Fraietta et al. combined ibrutinib with CTL019 for treatment of CLL, and the results demonstrated that Ibrutinib increased the expansion of CTL019 and decreased PD-1 and CD200 expression from CLL patients ex vivo. Moreover, they found that continuous ibrutinib treatment could enhance CTL019 efficacy in drug-resistant ALL and CLL mouse models. Ruella et al. [23] also added Ibrutinib to CTL019 for the treatment of mantle cell lymphoma; the results demonstrated that 80–100% of mice in the CTL019+ ibrutinib arm and 0–20% of mice in the CTL019 arm remained in long-term remission (p < 0.05). Prostaglandin E2 (PGE2) and adenosine are two other critical immunosuppressive mediators that are present in solid tumors. PGE2 and adenosine activate protein kinase A (PKA), which then inhibits T cell receptor (TCR) activation. This inhibition process requires PKA to localize to the immune synapse by binding to the membrane protein ezrin. Newick et al. [24] manufactured anti-mesothelin CAR T cells that co-expressed a small peptide called the “regulatory subunit I anchoring disruptor” (RIAD). RIAD can prevent the association of PKA with ezrin, thus eliminating the negative effects of PKA on TCR activation. Lenalidomide is a synthetic derivative of thalidomide, which has multifaceted immunomodulatory efficacies. The most outstanding function of lenalidomide is to restore and facilitate immune synapse formation between T cells and antigen presenting cells. Kuramitsu et al. [25] added lenalidomide to EGFRvIII (epidermal growth factor receptor variant III)-targeted CAR T cells against glioblastoma multiforme (GBM). An interesting and novel finding of their study was that lenalidomide enhanced immunological synapse formation between the effector cells and the target cells and then enhanced the persistent antitumor activity of CAR T cells. The interaction between HVEM (TNFRSF14) and BTLA (B and T lymphocyte attenuator) is lost in most follicular lymphomas due to an HVEM mutation. HVEM deficiency induces a tumor-supportive microenvironment. Boice et al. [26] modified anti-CAD19 CAR T cells to become in vivo micro-pharmacies for the local delivery of soluble HVEM, and these modified CAR-T cells showed enhanced therapeutic activity against xenografted lymphomas.

## Conclusion

In the above studies, in order to achieve better anti-tumor efficacy in the CAR T cell therapy, CAR T cells were modified to contain another single functional elements. The results demonstrate that incorporation of functional elements can significantly enhance the anti-tumor efficacy of CAR T cells. However, CAR T cells will encounter a more complicated and wicked tumor microenvironment after infuse into patients. Therefore, in order to make CAR T cells display the best anti-tumor capacity in clinic, it is better to simultaneously incorporate multiple functional elements into CAR T cells. For example, CAR T cells co-express PD1:CD28 chimera and IL12 can break the restriction imposed by immune inhibitory molecules and by the lack of cytokine signals. Expand CAR T cells with DMOG in vitro is also an option to make CAR T cells better overcome the hypoxic tumor microenvironment. By combining these approaches, CAR T cells can resist several adverse factors and finally we obtain more powerful CAR T cells.

## Abbreviations

CAR: chimeric antigen receptor
scFv: single chain fragment variable
Treg: regulatory T cells
PD1: programmed death 1
APC: antigen-presenting cell
TAM: tumor-associated macrophage
ICR: inverted cytokine receptor
CAIX: carbonic anhydrase IX
ccRCC: clear cell renal cell carcinoma
ADCC: antibody dependent cellular cytotoxicity
Onc.Ad: oncolytic adenovirus
HDAd: helper-dependent adenovirus
ROS: reactive oxygen species
PHD: prolyl hydroxylase domain
DMOG: dimethyloxalylglycine
CLL: chronic lymphocytic leukemia
BTK: bruton tyrosine kinase
ITK: inducible T cell kinase
PGE2: prostaglandin E2
PKA: protein kinase A
RIAD: regulatory subunit I anchoring disruptor
EGFRvIII: epidermal growth factor receptor variant III
GBM: glioblastoma multiforme
BTLA: B and T lymphocyte attenuator
